# Intraoperative OCT-Based Stromal Topography in Phototherapeutic Keratectomy

**DOI:** 10.3390/diagnostics16203345

**Published:** 2026-10-14

**Authors:** Vitus Grauvogl, Stefan Kassumeh, Wolfgang J. Mayer, Severin F. Mueller, Mehdi Shajari, Ludwig C. Hinske, Ines D. Nagel, Nikolaus Luft

**Affiliations:** 1 Department of Ophthalmology, Universitätsklinikum Augsburg, Stenglinstrasse 2, 86156 Augsburg, Germany; 2 Institute for Digital Medicine, Universitätsklinikum Augsburg, Stenglinstrasse 2, 86156 Augsburg, Germany; 3 Department of Ophthalmology, Universitätsklinikum München LMU, Mathildenstrasse 8, 80336 Munich, Germany; 4 Augenzentrum Kettenbrücke, Sennstrasse 1, 6020 Innsbruck, Austria; 5 Department of Ophthalmology, Goethe-University, Theodor-Stern-Kai 7, 60590 Frankfurt am Main, Germany; 6 Department of Anesthesiology, Universitätsklinikum München LMU, Marchioninistraße 15, 81377 München, Germany

**Keywords:** phototherapeutic keratectomy, epithelial basement membrane dystrophy, anterior segment optical coherence tomography, corneal higher-order aberrations, intraoperative corneal swelling

## Abstract

**Objectives:** This study aims to investigate whether anterior corneal curvature can be reliably measured by combined Placido–optical coherence tomography (OCT) during phototherapeutic keratectomy (PTK) despite intraoperative stromal swelling, and to quantify the immediate smoothing effect of PTK on anterior stromal higher-order aberrations (HOAs). **Methods:** In this prospective study, 20 eyes with epithelial basement membrane dystrophy (EBMD) undergoing PTK were examined using the MS-39© (CSO©) at four timepoints: preoperatively (t1), after epithelial debridement (t2), after PTK ablation (t3), and at 3-month follow-up (t4). Anterior curvature, corneal and stromal thickness, and epithelial thickness were analyzed across 17 corneal sectors within an 8 mm zone using two-way repeated-measures analysis of variance (ANOVA) (timepoint × sector). Anterior Zernike HOAs were computed globally, and the smoothing effect of PTK ablation was assessed by paired comparison of t2 and t3, both obtained on de-epithelialized stroma. **Results:** Mean central sagittal anterior curvature remained stable across intraoperative timepoints (t1: 44.27 ± 2.32 D; t2: 44.83 ± 2.75 D; t3: 45.01 ± 2.48 D; *p* = 0.490), despite substantial stromal swelling: Central corneal thickness increased by 114.4 ± 53.0 μm from t2 to t3 (*p* < 0.001), exceeding preoperative values. At 3-month follow-up, a significant flattening of −1.13 ± 1.38 D was observed (t3→t4; *p* = 0.002). Stromal HOA analysis revealed a significant smoothing effect of PTK ablation: total HOA root mean square (RMS) decreased from 2.28 ± 2.14 to 0.99 ± 0.46 μm (t2→t3; *p* = 0.001), with coma RMS showing the most consistent reduction (Δ = −0.38 ± 0.33 μm, *p* < 0.001; 19/20 eyes). Epithelial thickness distribution narrowed at t4 (interquartile range (IQR) −52.2%), consistent with epithelial remodeling after stromal surface regularization. **Conclusions:** Anterior corneal curvature remains stable during PTK despite intraoperative stromal swelling, supporting the feasibility of OCT-based intraoperative topographic assessment. In contrast, pachymetric readings are rendered unreliable by stromal hydration. Anterior stromal HOA, particularly coma RMS, may serve as a quantitative intraoperative marker of the smoothing effect achieved by PTK.

## 1. Introduction

Phototherapeutic keratectomy (PTK) is a well-established excimer laser procedure for the treatment of anterior corneal pathologies, including epithelial basement membrane dystrophy (EBMD) [1,2,3,4]. Formerly termed map-dot-fingerprint or Cogan microcystic dystrophy, this is a common disorder in which abnormal, thickened and duplicated basement membrane material impairs epithelial adhesion [4,5]. While many patients remain asymptomatic and thus need no treatment, others suffer recurrent painful erosions and reduced vision [4,5].

Management begins with lubricants and bandage contact lenses, escalating to anterior stromal micropuncture or mechanical debridement of the loosened epithelium. When these fail, superficial keratectomy and excimer laser PTK are next in line, with PTK particularly in cases with irregular astigmatism or subepithelial haze [4,5]. By selectively ablating diseased corneal tissue, PTK aims to restore a smooth anterior stromal surface, thereby improving visual acuity and reducing symptoms such as painful erosive episodes [2,5,6].

A critical challenge in PTK remains the determination of the surgical endpoint. During conventional PTK, the surgeon removes the corneal epithelium mechanically and subsequently applies excimer laser pulses to exposed corneal stroma. In certain cases, adjunctive use of masking fluids (e.g., balanced salt solution, hydroxypropyl methylcellulose, or hyaluronate) can foster smoothing of the corneal stromal surface (“wet PTK”) [4]. Intraoperatively, the surgeon must visually judge whether residual stromal surface irregularity remains, since this determines whether additional ablation is warranted [2]. This leads to variability and reduced reproducibility, especially in complex cases where residual irregularities may not be immediately apparent during surgery.

Objective intraoperative assessment of corneal surface regularity represents a significant advance for PTK. Zhou et al. demonstrated that swept-source optical coherence tomography (OCT)-based corneal topography measures stromal surface curvature immediately after epithelial debridement, reporting an anterior surface steepening attributable to the loss of the epithelial lenticular effect [7]. This landmark study showed excellent repeatability of stromal surface topography and established that corneal swelling following de-epithelialization did not significantly affect the measured curvature parameters. However, this study was limited to the pre-ablation state after epithelial removal and did not examine whether curvature measurements remained interpretable after the additional perturbation of excimer laser ablation and the associated corneal changes. This distinction is clinically relevant. The post-ablation curvature needs to be evaluated by the surgeon to determine whether the therapeutic goal has been achieved.

The feasibility of such post-ablation measurements is further complicated by corneal stromal swelling. Once the epithelial barrier is removed, the anterior stroma rapidly absorbs fluid, leading to significant increases in corneal thickness [8]. This swelling is not uniform across the stromal depth; the anterior stroma, with its lower degree of collagen cross-linking and higher concentration of dermatan sulfate proteoglycans, exhibits greater swelling capacity than the more tightly interwoven posterior layers [8,9]. Dupps and Roberts demonstrated in a cadaver model that biomechanical changes following keratectomy—including peripheral stromal thickening of up to 57 μm—lead to significant anterior surface flattening [10]. Whether such anterior stromal edema distorts curvature measurements to a degree that renders them clinically unreliable during PTK has not been systematically investigated.

Several groups have explored intraoperative imaging during excimer laser surgery. Vinciguerra and Camesasca introduced custom PTK with intraoperative topography-based corneal aberrometry, demonstrating its feasibility in highly aberrated eyes [11]. More recently, the same group reported long-term outcomes of sequential custom therapeutic keratectomy (SCTK) using intraoperative tomographic monitoring between ablation steps for granular corneal dystrophy [12]. Siebelmann et al. described the use of microscope-integrated OCT during PTK for real-time thickness monitoring [13]. However, none of these studies systematically validated the reliability of anterior curvature measurements after excimer laser ablation on a de-epithelialized and edematous cornea. Moreover, the clinical significance of epithelial remodeling (whereby the epithelium compensates for stromal irregularities by varying its thickness [14,15]) underscores the importance of measuring the true stromal surface curvature intraoperatively, before epithelial regrowth masks residual irregularities.

Recent advances in combined traditional topography and anterior-segment OCT now allow simultaneous high-resolution mapping of corneal thickness, curvature, and elevation across the entire corneal surface. The MS-39© (CSO©, Florence, Italy) combines Placido disk topography with spectral-domain OCT, provides separate quantification of epithelial and stromal layers, and has been validated for corneal assessment in both normal and keratoconic eyes [16,17].

Therefore, the aim of this prospective study was to investigate whether anterior corneal curvature can be reliably measured by combined Placido–OCT during PTK—specifically after epithelial debridement and after excimer laser ablation. We hypothesized that while stromal thickness increases substantially after epithelial removal and PTK ablation, anterior curvature measurements remain stable and clinically interpretable. Confirmation of this hypothesis would open the possibility of using intraoperative tomography to objectively assess corneal surface irregularity, enabling the surgeon to determine whether additional ablation is needed during wet-PTK or transepithelial PTK procedures. This could be achieved through intraoperative measurement of wavefront-based regularity metrics, which are performed after each ablation pass and compared with a predefined endpoint.

## 2. Materials and Methods

In this prospective observational study performed at the University Eye Hospital of the Ludwig Maximilian University (LMU), consecutive patients scheduled for PTK due to EBMD were enrolled. Informed consent was obtained from all participants, and all study-related procedures were conducted in accordance with the ethical standards of the institutional ethics committee (vote number 22-1001) as well as in accordance with the Declaration of Helsinki. A total of 20 eyes of 19 patients (mean age 52.1  ±  17.7 years; range 28–87; 10 female, 9 male) were included. Inclusion criteria were a clinical indication for PTK, age ≥ 18 years, and the ability to provide informed consent. Exclusion criteria included previous corneal surgery, contact lens wear within 4 weeks, and other corneal disease interfering with measurements such as keratoconus.

### 2.1. Study Protocol and Timepoints

Corneal tomography was acquired at three timepoints during each PTK procedure and additionally at follow-up: (t1) preoperatively with intact epithelium; (t2) immediately after manual epithelial debridement; (t3) immediately after completion of the PTK ablation; as well as (t4) at 3-month follow-up with re-epithelialized cornea. All acquisitions were obtained using the same device and examination protocol, and all patients attended the scheduled three-month follow-up.

### 2.2. Corneal Imaging

All participants underwent high-resolution anterior segment spectral-domain OCT combined with Placido disk-based topography (MS-39©, CSO©, Florence, Italy). Adequate image acquisition quality was ascertained by the onboard software’s scan quality indicator in all cases. The device was maintained under the manufacturer’s service schedule, was used with unchanged software settings throughout the study period and does not require user-side calibration. The MS-39© provides simultaneous measurement of corneal thickness (total, epithelial, and stromal), curvature (sagittal, tangential, and Gaussian for both anterior and posterior surfaces), elevation (anterior, posterior, and stromal), and refractive power maps. Repeatability of MS-39 tomography and epithelial thickness mapping has been established in independent cohorts [16,17]. Raw data were exported using the device’s comma-separated values (CSV) export function for each scan. Each export file contained measurements across 31 concentric rings (radii 0.0–6.0 mm in 0.2 mm steps) with 256 equidistant hemimeridians for each ring (angular spacing 1.41°), yielding 7936 measurement points per parameter per scan.

### 2.3. Phototherapeutic Keratectomy

PTK was performed by experienced cornea specialists (W.J.M., N.L., M.S.) under topical tetracaine anesthesia using a MEL 90 excimer laser (Carl Zeiss Meditec AG©, Jena, Germany) with a standardized epithelium-off protocol: planar phototherapeutic profile, 8.0 mm optical treatment zone, 0 mm transition zone, programmed ablation depth 12 µm delivered in a single pass, no topography- or wavefront-guided customization. The ablation depth was fixed for all eyes to standardize the intervention within this prospective protocol, and the MS-39 measurements were not used to adjust the treatment. Centration was achieved on the fixation target with active eye tracking; ablation was performed through a thin film of balanced salt solution applied to the treatment zone (wet-PTK technique). Tomographic acquisition at t2 followed epithelial debridement with a hockey knife, and at t3 immediately after ablation. For both acquisitions, the eyelid speculum was removed, and patients sat up, left the laser bed, and were seated at the MS-39, where they fixated on its internal target. Blinking was permitted, no additional fluid or lubricant was applied before acquisition, and the topical tetracaine anesthesia minimized reflex tearing.

Postoperatively, a bandage contact lens was retained until complete re-epithelialization. Patients were instructed to instill levofloxacin 0.5% four times daily for one week and dexamethasone 0.1% four times daily, tapered by one instillation per week over four weeks. Adherence was not formally recorded.

### 2.4. Data Processing

Raw CSV data from all 80 acquisitions (20 eyes at four timepoints) were parsed into a unified long-format dataset using custom Python scripts (Python 3.9.6, Python Software Foundation, Wilmington, DE, USA, https://www.python.org; NumPy 2.0.2, https://numpy.org; pandas 2.3.3, https://pandas.pydata.org). Hemimeridian angles for left eyes (OS) were horizontally mirrored to right-eye (OD) convention using the transformation (180  −  α) mod 360° to ensure anatomically consistent nasal–temporal orientation across both eyes.

For spatial analysis, the corneal surface was divided into 17 sectors within the central 4.0 mm zone: one central sector (radius  ≤  2.0 mm), eight paracentral sectors (2.0  <  radius  ≤  3.0 mm), and eight peripheral sectors (3.0  <  radius  ≤  4.0 mm). The paracentral and peripheral zones were each subdivided into eight angular sectors of 45° (superior, superonasal, nasal, inferonasal, inferior, inferotemporal, temporal, superotemporal) following anatomical convention for the right eye.

### 2.5. Artifact Filtering

Values were regarded as artefactual when they arose either from acquisition (lid and lash shadowing, eye movement, and at t2/t3 the absent regular tear film over de-epithelialized stroma, which degrades the anterior boundary signal) or from segmentation failure at the periphery of the scanned zone, rather than from corneal anatomy. Because sector means differ by only a few micrometers, such values were removed rather than corrected; no imputation was performed. Remaining values outside predefined plausibility limits (corneal thickness 200–1200 µm; epithelial thickness 0–100 µm; stromal thickness 200–1000 µm) were set to missing, as were values beyond median ± 5 × median absolute deviation (MAD) within each measurement type, radius and timepoint. The MAD-based threshold, being insensitive to the outliers it targets, allowed a conservative factor of 5 (≈3.7 standard deviation (SD)) that removes clear measurement failures while preserving the biological variability expected after debridement and ablation.

### 2.6. Corneal Thickness Analysis

Stromal thickness was analyzed at all four timepoints. Intraoperative stromal swelling was quantified as the difference in corneal thickness between consecutive timepoints (t2 − t1 and t3 − t2). Because no intact epithelium was present at t2 and t3, a composite approach was used: stromal thickness at t1 and t4 (with intact epithelium) was compared with total corneal thickness at t2 and t3 (which equals stromal thickness in the absence of epithelium) to track stromal changes across all four timepoints. Epithelial thickness was analyzed at t1 and t4 only. The interquartile range (IQR) of the mean epithelial thickness was calculated to assess epithelial homogenization after PTK.

### 2.7. Higher-Order Aberration Analysis

Zernike coefficients (orders 3–7) were extracted from the MS-39© onboard wavefront analysis for a 6.0 mm analysis diameter centered on the corneal vertex. For each eye and timepoint, root mean square (RMS) values were computed for clinically relevant aberration groups: total higher-order aberrations (HOAs) coma, trefoil and spherical aberration. Timepoints (t1-4) were assigned chronologically according to the aforementioned study protocol.

### 2.8. Statistical Analysis

Statistical analysis was performed using Python 3.9.6 with pingouin 0.5.5 (https://pingouin-stats.org) and SciPy 1.13.1 (https://scipy.org). Distribution normality was assessed using the Shapiro–Wilk test. Two-way repeated-measures analysis of variance (RM-ANOVA) with factors timepoint (4 levels) and sector (17 levels) was conducted for each of the 14 corneal measurement parameters, using a complete-case approach requiring all 68 cells (4  ×  17) per subject. Greenhouse–Geisser correction (pGG) was applied where sphericity was violated. Effect sizes are reported as generalized eta-squared (η_G_^2^).

For HOA analyses, one-way RM-ANOVA or Friedman tests (depending on normality) were used to assess the timepoint effect on each RMS metric, with Bonferroni-corrected post hoc pairwise comparisons. Paired *t*-tests or Wilcoxon signed-rank tests were used for planned pairwise comparisons (t1 vs. t4; t2 vs. t3). Effect sizes are reported as Cohen’s *d*. For all sector-level pairwise comparisons, false discovery rate (FDR) correction was applied for multiple testing. Exploratory Spearman rank correlations related preoperative (t1) HOA RMS to the HOA reduction from t2 to t3. To account for the one patient contributing both eyes, the main analyses were repeated after excluding the second-treated eye. A two-tailed *p*-value of <0.05 was considered statistically significant.

### 2.9. Use of Generative Artificial Intelligence

Claude Sonnet 5.5 (Anthropic PBC, San Francisco, CA, USA) was used for language editing of the manuscript. All output was reviewed, verified, and edited by the authors. Generative AI was not used for data interpretation or for drawing scientific conclusions.

## 3. Results

MS-39© tomography acquisitions were successfully obtained at all four timepoints in all patients, including the de-epithelialized cornea at t2 and the edematous post-ablation cornea at t3. Excluding the second eye of the bilaterally treated patient did not change any of the main findings.

### 3.1. Stromal Thickness Changes and Intraoperative Corneal Swelling

As depicted in Figure 1, mean central corneal thickness was 586.1 ± 40.4 μm at baseline (t1), decreased to 518.8 ± 44.7 μm after epithelial debridement (t2), increased again to 633.2 ± 52.3 μm after PTK ablation (t3), and returned to 565.9 ± 29.7 μm at 3-month follow-up (t4). Hence, at t3, corneal thickness far exceeded preoperative values despite excimer laser ablation of stromal tissue programmed to 12 µm. At 3 months, net central stromal thickness loss was 13.2 ± 23.9 μm (*p* = 0.027, *d* = − 0.55).

Corneal thickness was analyzed by two-way repeated-measures ANOVA (timepoint × sector). The timepoint of measurement had the strongest influence: the four timepoints differed clearly from one another (*p* < 0.001, ηG^2^ = 0.430). The 17 sectors also differed, but by a smaller margin (*p* < 0.001, ηG^2^ = 0.372), reflecting an increase in thickness from center to periphery. The regional pattern shifted only slightly over time (interaction *p* = 0.028, ηG^2^ = 0.017).

Stromal thickness followed the same time course (*p* < 0.001, ηG^2^ = 0.459, see Figure 2). Here, the regional dependence did not reach the significance threshold (*p* = 0.052, ηG^2^ = 0.014).

### 3.2. Epithelial Thickness Changes

Mean epithelial thickness across the central 8 mm zone was 54.90  ±  5.06 μm at t1 and 51.64  ±  4.42 μm at t4 (*p* = 0.011). The within-eye interquartile range of epithelial thickness decreased from 12.37 ± 7.06 µm at t1 to 5.91 ± 3.80 µm at t4 (*p* < 0.001), indicating a narrower distribution of epithelial thickness values and, thus, regularization at three months postoperatively, as well as a physiological superior-to-inferior thickness gradient (Figure 3).

### 3.3. Anterior Curvature Changes

Mean central sagittal anterior curvature was 44.27 ± 2.32 diopters (D) at baseline (t1), 44.83 ± 2.75 D after epithelial debridement (t2), 45.01 ± 2.48 D after PTK ablation (t3), and 43.88 ± 1.85 D at 3-month follow-up (t4). Only the anterior curvature change in t3 compared to t4 reached statistical significance (Δ = −1.13 ± 1.38 D, *p* = 0.002, *d* = −0.82) (Table 1, Figure 4).

Sagittal anterior curvature remained measurable throughout the procedure without a detectable time-related change: averaged over all sectors, curvature at the four timepoints could not be distinguished statistically (pGG = 0.490, ηG^2^ = 0.001). What did separate the measurements was location: curvature differed consistently between the 17 sectors (*p* < 0.001, ηG^2^ = 0.176). A weak dependence of the sector pattern on timepoint was detectable (interaction *p* = 0.042, ηG^2^ = 0.016), meaning that individual areas shifted slightly relative to one another while the overall curvature was preserved.

Tangential and Gaussian curvature, which are derived from the local rather than the apex-referenced surface geometry, responded more strongly. Tangential curvature changed significantly between timepoints (pGG < 0.001, ηG^2^ = 0.169), and the change was not uniform across the cornea (timepoint-by-sector interaction pGG < 0.001, ηG^2^ = 0.238). Gaussian curvature showed the same behavior in attenuated form (time effect pGG < 0.001, ηG^2^ = 0.066; and interaction pGG < 0.001, ηG^2^ = 0.089).

### 3.4. Anterior Higher-Order Aberrations

Mean total HOA RMS increased from 1.29 ± 1.42 μm at baseline (t1) to 2.28 ± 2.14 μm after epithelial debridement (t2), decreased to 0.99 ± 0.46 μm after PTK ablation (t3), and measured 0.83 ± 0.81 μm at the 3-month follow-up (t4).

The comparison between t2 and t3—both measured on the de-epithelialized stroma—showed a significant reduction in total HOA RMS (Δ = −1.29 ± 2.09 μm, *p* = 0.001), with 17 of 20 eyes showing a decrease (Figure 5). Coma RMS showed the most consistent reduction (Δ = −0.38 ± 0.33 μm, *p* < 0.001, 19/20 eyes reduced) (Table 2). The reduction from t2 to t3 was not significantly associated with preoperative total HOA RMS (Spearman ρ = 0.36, *p* = 0.115).

Comparing baseline (t1) to 3-month follow-up (t4), total HOA RMS decreased significantly (Δ = −0.46 ± 0.75 μm, *p* = 0.002). Individual HOA components (coma, trefoil, spherical aberration) did not reach significance for the t1–t4 comparison.

## 4. Discussion

### 4.1. Anterior Curvature Stability

In this study, anterior sagittal curvature remained stable throughout the intraoperative phases of PTK, despite substantial stromal swelling during surgery (repeated-measures ANOVA revealed no significant timepoint effect for central sagittal anterior curvature; *p* = 0.490, ηG^2^= 0.001). Neither the transition from intact cornea to debrided stroma (t1 to t2: +0.56 ± 1.81 D, *p* = 0.191) nor the subsequent excimer ablation step (t2 to t3: +0.18 ± 1.11 D, *p* = 0.494) induced a significant change in anterior curvature. This finding is particularly striking given that stromal swelling between t2 and t3 averaged +114.4 ± 53.0 µm (*p* < 0.001). These results suggest that OCT-based anterior curvature measurement during PTK provides reliable topographic information even in the presence of marked stromal edema.

Our data build on and extend the work of Zhou et al. [7], who demonstrated the feasibility of OCT-based stromal topography after epithelial debridement alone. They reported a steepening of +1.28 D upon epithelial removal, a value broadly consistent with our observed +0.56 D change at t2, though the difference may reflect variations in epithelial thickness profiles and measurement techniques. Critically, while Zhou et al. confined their analysis to the post-debridement state, the present study extends intraoperative stromal topography to the post-ablation phase, demonstrating for the first time that anterior curvature remains stable even after excimer laser application and despite the ensuing stromal hydration. Previous work by Vinciguerra and Camesasca established the concept of intraoperative topography-guided PTK [11], and Siebelmann et al. utilized microscope-integrated OCT during PTK for real-time pachymetric monitoring [13]. However, neither study systematically validated whether the curvature data obtained intraoperatively remained distortion-free in the setting of stromal swelling. Our findings provide this missing validation.

The preservation of anterior curvature despite massive axial stromal expansion can be understood through the biomechanical architecture of the anterior stroma. Müller et al. demonstrated that the anterior corneal stroma is characterized by a dense meshwork of interwoven collagen lamellae, in contrast to the more loosely arranged posterior layers [8]. This structural arrangement confers greater resistance to tangential displacement, effectively constraining the anterior surface geometry even as the stroma swells in the axial direction. Notably, Dupps and Roberts predicted in their cadaver-based biomechanical model that peripheral stromal thickening of 57 µm could produce a flattening of up to 6.3 D after keratectomy [10]. The discrepancy between their predicted curvature distortion and our observed stability likely reflects fundamental differences between ex vivo and in vivo conditions: in the living cornea, intact limbal support and intraocular pressure provide boundary constraints that limit peripheral stromal expansion and preserve the overall corneal contour.

The only significant curvature change occurred between t3 and t4 (−1.13 ± 1.38 D, *p* = 0.002), representing a postoperative flattening at 3 months that is consistent with the expected mild hyperopic shift following superficial ablation [10,18,19,20]. This delayed change likely reflects stromal remodeling and epithelial hyperplasia rather than an intraoperative measurement artifact and will be discussed further in the context of refractive outcomes below.

### 4.2. Stromal Swelling

Pachymetric data revealed a striking degree of intraoperative stromal swelling. Following epithelial removal, central corneal thickness decreased from 586.1 ± 40.4 µm to 518.8 ± 44.7 µm (−67.3 ± 39.6 µm, *p* < 0.001), consistent with the loss of the epithelial layer. After PTK, however, corneal thickness rose sharply to 633.2 ± 52.3 µm (+114.4 ± 53.0 µm from t2, *p* < 0.001), exceeding preoperative values despite the combined removal of approximately 55 µm of epithelium and 12 µm of stromal tissue. This magnitude of stromal hydration is comparable to the +115 µm increase reported during corneal cross-linking with hypo-osmolar riboflavin solutions [21], yet in the present study it occurred without the application of any hypo-osmolar agent, driven solely by exposure of the de-epithelialized stroma to balanced salt solution irrigation during excimer laser treatment. Two-way repeated-measures ANOVA confirmed a large main effect of timepoint on corneal thickness (*p* < 0.001, ηG^2^ = 0.430), and the significant timepoint × sector interaction (*p* = 0.028, ηG^2^ = 0.017), though small in effect size, indicated subtle regional differences in the swelling response. These regional variations likely reflect the known anterior-to-posterior gradient in stromal hydration susceptibility, which has been attributed to differences in collagen lamella organization and proteoglycan distribution between the anterior and peripheral stroma [8,9].

To our knowledge, this is the first study to provide spatially resolved, sector-based mapping of stromal swelling during PTK using polar heatmaps derived from intraoperative OCT topography. The composite stromal thickness maps (Figure 2), which used stromal thickness at t1 and t4 and total corneal thickness at the de-epithelialized timepoints t2 and t3, demonstrated a largely uniform swelling pattern across all 17 sectors. This spatial uniformity was corroborated by the two-way RM-ANOVA for stromal thickness, which showed a large timepoint effect (*p* < 0.001, ηG^2^ = 0.459) but no significant timepoint × sector interaction (*p* = 0.052), suggesting that the stroma swells in a relatively homogeneous fashion. By the three-month follow-up, net corneal thinning of −20.2 ± 21.6 µm (*p* < 0.001) and net stromal thinning of −13.2 ± 23.9 µm (*p* = 0.027) confirmed that the transient intraoperative swelling had fully resolved, with the residual thickness reduction attributable to the ablation and epithelial remodeling.

The clinical implication of these findings is specific to pachymetry: intraoperative thickness measurements obtained after epithelial removal cannot be used to reliably estimate residual stromal thickness during PTK, whereas anterior curvature and surface-regularity metrics remain interpretable under the same hydration conditions and can be used as objective parameters regarding the success of the smoothening effect. A swelling magnitude of over 100 µm renders any thickness-based safety threshold—such as the commonly cited 250 µm residual stromal bed [3,22] essentially uninterpretable without correction for hydration status. This reinforces the value of curvature-based assessment as a more robust intraoperative parameter.

### 4.3. Epithelial Remodeling

Direct evidence for epithelial remodeling was found in the thickness profiles obtained at baseline and three months postoperatively. Mean epithelial thickness decreased modestly from 54.90 ± 5.06 µm at t1 to 51.64 ± 4.42 µm at t4, but the more telling change occurred in the distribution: the interquartile range narrowed from 12.37 µm to 5.91 µm at t4 (a 52.2% reduction). The polar heatmaps corroborated that at baseline, the epithelial map displayed the characteristic pattern of focal central-to-inferior thickening that is well recognized in EBMD, where the epithelium compensates for underlying stromal irregularity by filling in surface depressions [23]. After PTK, this compensatory pattern gave way to a more uniform thickness profile (Figure 3). These findings align closely with earlier results using the same MS-39© platform. Epithelial thickness IQR proved to be a diagnostic marker for EBMD, with significant normalization of IQR after PTK at three months [24]. This reinforces the interpretation that PTK, by smoothing the anterior stromal surface, removes the stimulus for compensatory epithelial thickening and allows the epithelium to assume a more physiological configuration [15].

### 4.4. HOA Analysis

Beyond curvature map discrepancies, higher-order aberration analysis offered a complementary lens through which to quantify the stromal surface changes induced by PTK. Because both the post-debridement (t2) and post-PTK (t3) measurements were obtained on bare stroma—free of epithelial masking—the t2-to-t3 comparison provided a direct assessment of the ablation’s smoothing effect. Total HOA RMS decreased from 2.28 ± 2.14 to 0.99 ± 0.46 µm (Δ = −1.29 µm; *p* = 0.001), with 17 of 20 eyes showing a reduction (see Figure 5). Among individual components, coma RMS demonstrated the most consistent response, followed by trefoil RMS and spherical aberration (see Table 2). Notably, while Zhou et al. reconstructed stromal topography from swept-source OCT data, they did not compute wavefront-derived metrics from these surfaces [7]. The present findings suggest that anterior stromal HOA—particularly coma RMS, given its near-universal reduction and large effect size—may serve as a quantitative marker of the immediate smoothing achieved by PTK [25].

### 4.5. Rationale for Intraoperative Assessment

Intraoperative assessment and postoperative follow-up answer two different questions. The ablation decision must be made while the patient is still on the table; in routine PTK, no staged re-treatment is planned, so the intraoperative scan coincides with the only moment at which the surgeon can still act. It is also the only window in which the stromal surface itself is accessible, because the regenerating epithelium compensates for residual stromal irregularity by local thickening and thereby masks what needs to be judged [14,15]. Postoperative follow-up answers the second question, documenting how the eye responds to the state achieved intraoperatively and whether the smoothing recorded in bare stroma persists. Demonstrating that intraoperative measurement is reliable is the precondition for the subsequent question of whether titrating ablation to such measurements improves outcomes [11,12].

### 4.6. Limitations

Several limitations of this study merit consideration beyond those already addressed in context. First, the sample size of 20 eyes from 19 patients constrains statistical power. Although the consistent directionality of our findings across multiple independent metrics lends confidence to the observed patterns, larger cohorts will be needed to confirm the effects. Second, the validity of Placido-based topography on de-epithelialized stroma deserves scrutiny. The MS-39 relies on specular reflection from the corneal surface for its anterior curvature measurements; on bare stroma without a smooth tear film, reflection quality may be compromised compared to measurements on intact epithelium. While the device’s integrated quality indicator accepted all intraoperative scans, this does not guarantee equivalent measurement accuracy. The OCT-derived thickness and elevation measurements are likely more robust in this setting, but our curvature data at t2 and t3 should be interpreted with this caveat in mind. Likewise, part of the HOA reduction from t2 to t3 may reflect differences in surface hydration or residual fluid after wet-PTK rather than tissue removal alone. Third, the time interval between epithelial debridement and the t2 scan, as well as between ablation completion and the t3 scan, was neither standardized nor recorded, although all acquisitions were completed within minutes. Given that stromal hydration is a time-dependent process, this variability may have contributed to the inter-patient variance in swelling magnitude observed at these timepoints. Fourth, repeatability was not assessed within the present cohort. A single acquisition was obtained at each timepoint, and the published repeatability data for this device [16,17] were derived from epithelialized corneas; they therefore do not necessarily transfer to measurements on bare stroma, where the specular reflection used for Placido-based curvature is degraded. Fifth, the design includes no untreated control group: each eye served as its own comparator across four timepoints, which eliminates between-subject variability, but does not allow one to reference the observed changes against an eye not undergoing PTK.

Future studies should aim to validate these findings in larger, multi-indication cohorts that include stromal dystrophies and corneal scars, with longer follow-up to capture the full trajectory of epithelial remodeling and to assess recurrence rates and refractive stability. The integration of functional outcome measures will be essential to translate morphological observations into clinically actionable benchmarks. From a technological perspective, real-time computation of higher-order aberrations from intraoperative tomographic data could enable the surgeon to titrate ablation depth to a predefined optical endpoint rather than relying on fixed protocols, a concept that aligns with the broader trend toward personalized, feedback-guided corneal surgery.

## 5. Conclusions

In conclusion, this prospective study demonstrates that intraoperative OCT can capture the full morphological sequence of PTK in a single surgical session, revealing immediate stromal swelling after epithelial debridement, curvature preservation despite tissue removal, and a smoothing effect on higher-order aberrations that persists into the postoperative period. These findings support OCT-based stromal topography, acquired at discrete timepoints during surgery, as a feasible and informative tool for assessing the stromal surface in PTK; its use for guiding the ablation remains to be tested.

## Figures and Tables

**Figure 1:**
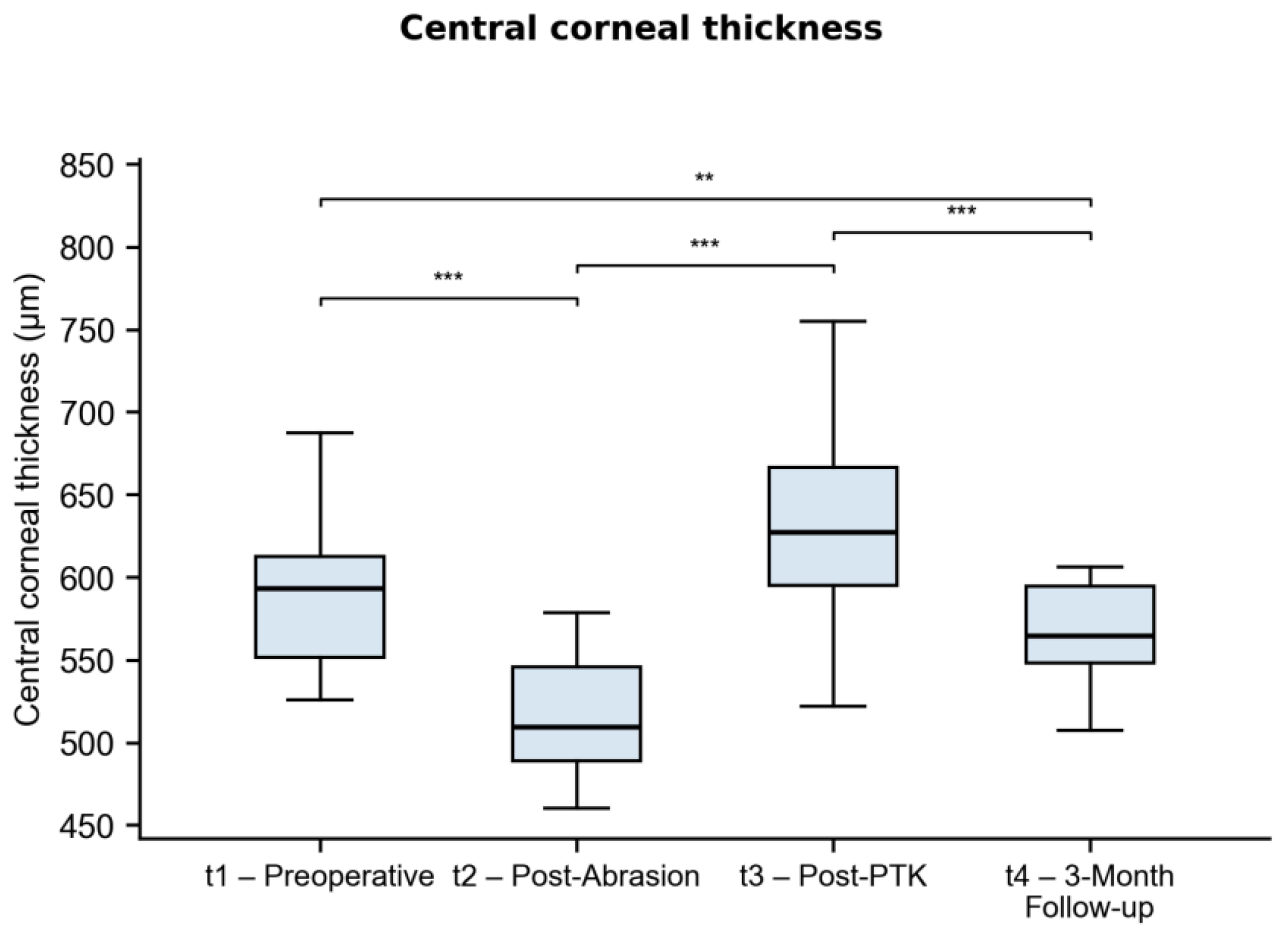
Central corneal thickness across surgical timepoints. Significance brackets indicate Bonferroni-corrected paired *t*-tests (*** *p* < 0.001, ** *p* < 0.01). Central corneal thickness increased from post-debridement (t2) to post-phototherapeutic keratectomy (PTK) (t3) despite tissue ablation, reflecting intraoperative stromal swelling, and was significantly reduced at 3-month follow-up compared with baseline (*p* = 0.004).

**Figure 2:**
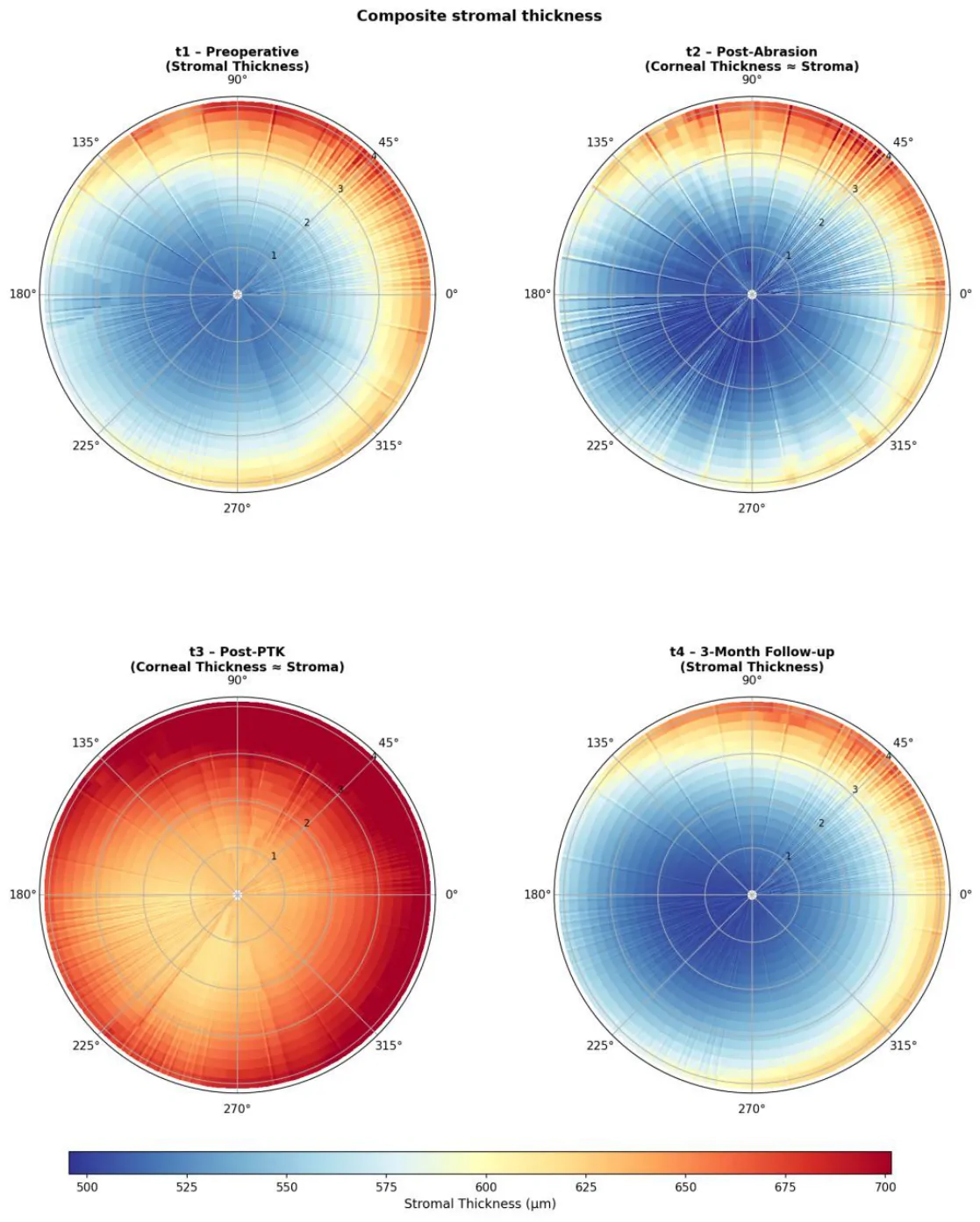
Composite polar heatmaps of stromal thickness (µm) averaged across all patients (radius ≤ 4 mm). At t1 (preoperative) and t4 (3-month follow-up), stromal thickness was measured directly; at t2 (post-abrasion) and t3 (post-PTK), total corneal thickness was used as a surrogate for stromal thickness, as the epithelium was absent. Color scale is shared across all four panels. Central thinning at t3 reflects the PTK ablation zone, while t4 shows partial stromal recovery.

**Figure 3:**
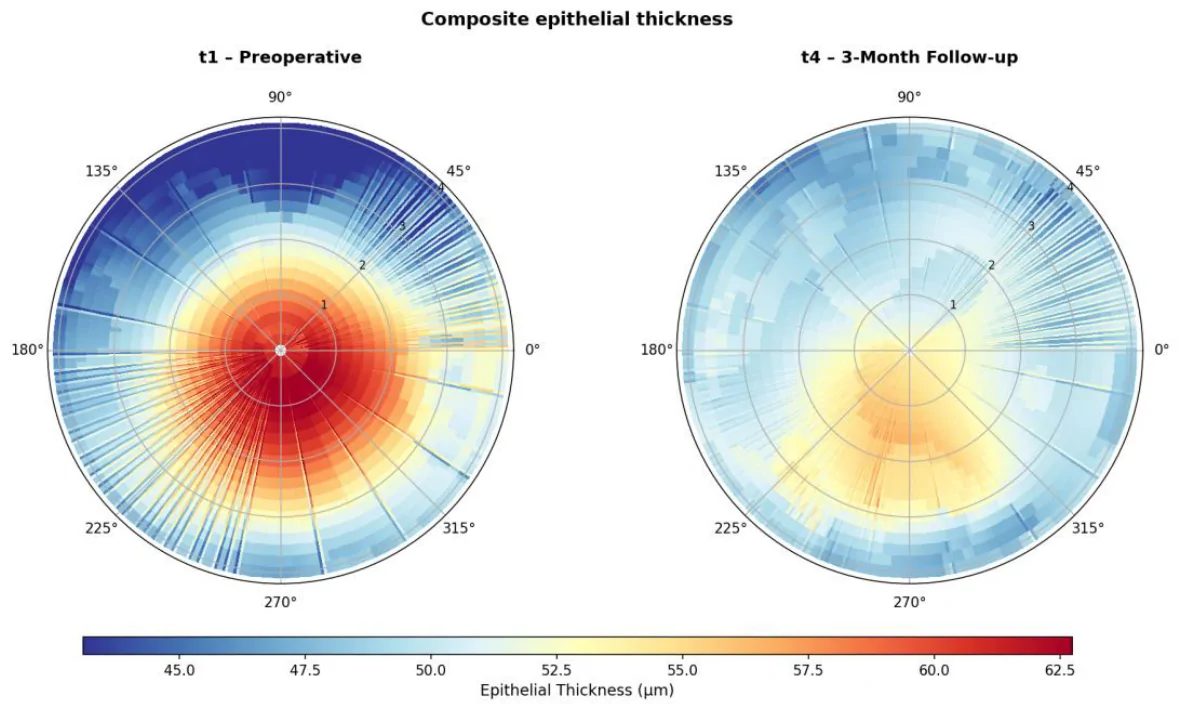
Polar heatmaps of mean epithelial thickness (µm) across all patients (radius ≤ 4 mm) at t1 (preoperative) and t4 (3-month follow-up). Timepoints t2 and t3 were excluded because the epithelium was absent after abrasion/PTK, rendering segmentation unreliable. At t1, a typical epithelial profile is seen with central–inferior thickening. At t4, the epithelial distribution appears more uniform with reduced central prominence, suggesting epithelial remodeling following PTK.

**Figure 4:**
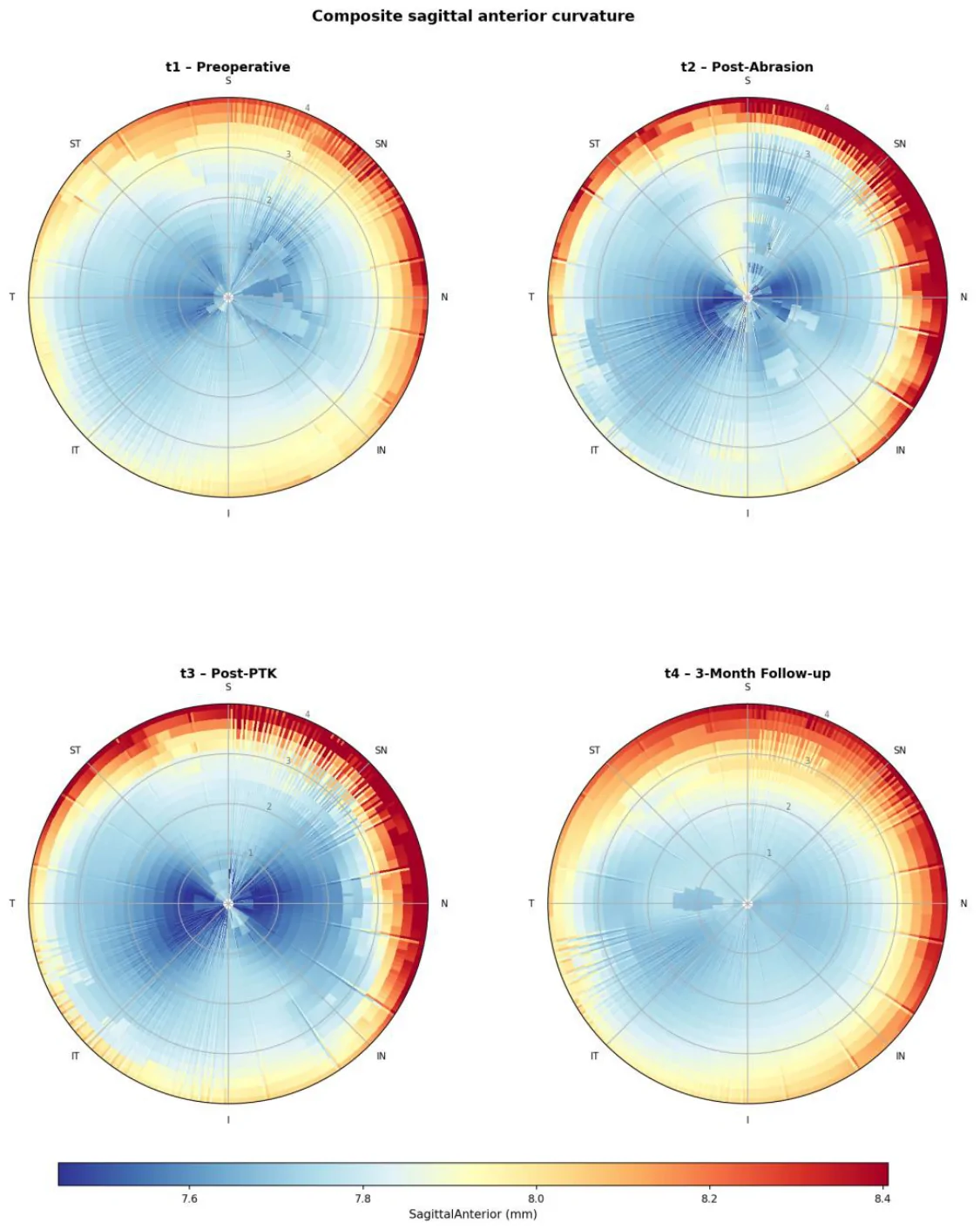
Polar heatmaps of mean sagittal anterior curvature radius (mm) across all patients (radius ≤ 4 mm) at four timepoints: t1 (preoperative), t2 (post-abrasion), t3 (post-PTK), and t4 (3-month follow-up). Note the peripheral steepening at t2 and t3 following epithelial removal and stromal ablation, with partial recovery at t4. Angular sectors are labeled according to right-eye (OD) convention (N = nasal, T = temporal); left eyes were mirrored accordingly.

**Figure 5:**
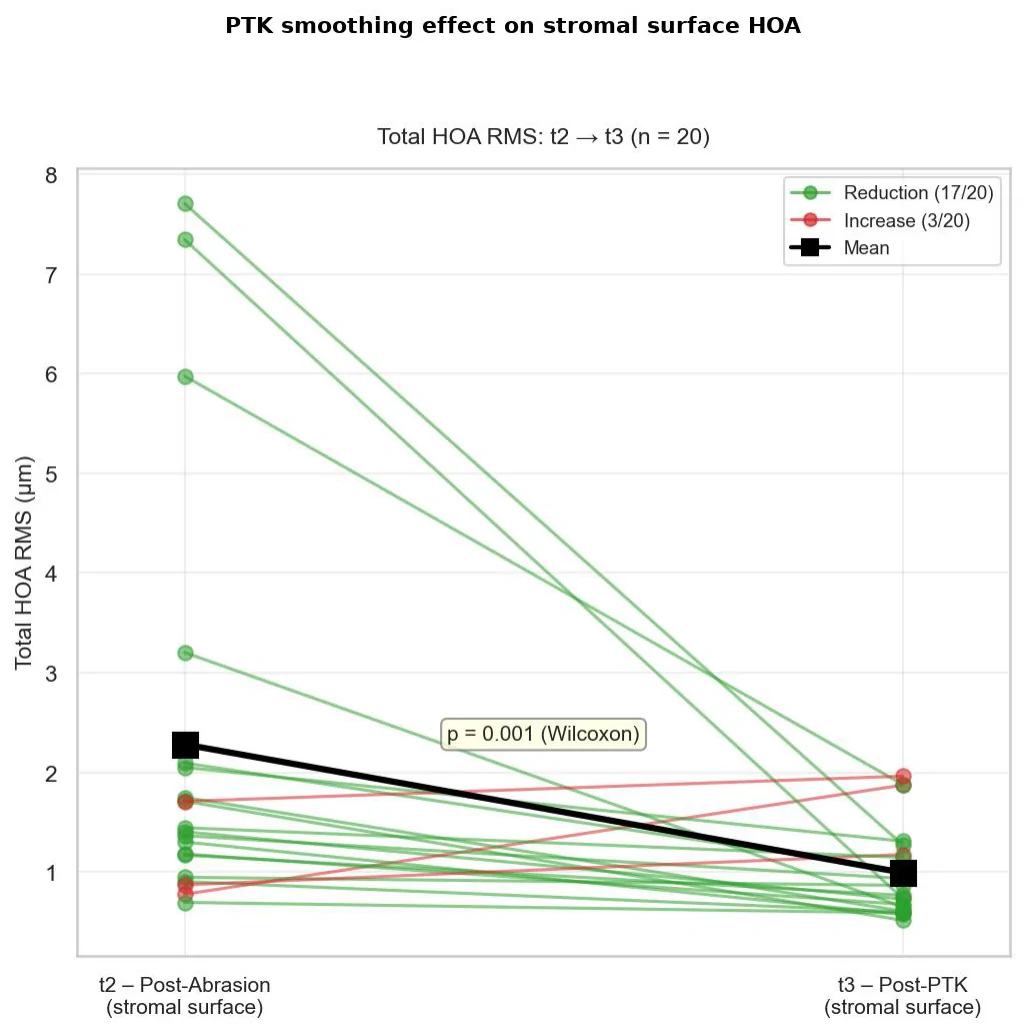
Spaghetti plot showing the change in total higher-order aberration (HOA) root mean square (RMS, µm) of the anterior stromal surface from t2 (post-abrasion) to t3 (post-PTK). Each line represents one eye (*n* = 20). Green lines indicate a reduction in HOA RMS (17/20 eyes, 85%); red lines indicate an increase (3/20 eyes, 15%). Black diamonds denote the group mean. The reduction from t2 to t3 was statistically significant (*p* < 0.001, Wilcoxon signed-rank test), confirming the smoothing effect of PTK on stromal surface irregularities.

**Table 1:** Two-way repeated-measures analysis of variance (ANOVA) for anterior curvature parameters. Main effects of timepoint (t1–t4) and sector (17 corneal sectors within 4 mm radius), and their interaction are shown. F = F-statistic; *p*-values are Greenhouse–Geisser-corrected (pGG). Effect size is reported as generalized eta-squared (ηG^2^).

Parameter	Effect	F	*p*_GG_	η_G_^2^
Sagittal Anterior	Timepoint	0.73	0.490	0.001
	Sector	19.78	<0.001	0.176
	Interaction	2.61	0.042	0.016
Tangential Anterior	Timepoint	21.50	<0.001	0.169
	Sector Interaction	44.18 13.76	<0.001 <0.001	0.484 0.238
Gaussian Anterior	Timepoint	20.62	<0.001	0.066
	Sector	46.68	<0.001	0.480
	Interaction	6.19	<0.001	0.089

**Table 2:** Higher-order aberration smoothing effect of PTK ablation. Anterior Zernike higher-order aberration (HOA) metrics (orders 3–7, 6.0 mm analysis diameter) are compared between t2 (post-debridement) and t3 (post-PTK), both measured on de-epithelialized stroma. Δ = mean paired difference; *p* = Wilcoxon signed-rank test; *d* = Cohen’s *d*; RMS = root mean square; SD = standard deviation; Reduced = number of eyes showing a decrease. *n* = 20 eyes.

Metric	t2 (Mean ± SD)	t3 (Mean ± SD)	Δ	*p*	*d*	Reduced
Total HOA RMS	2.28 ± 2.14	0.99 ± 0.46	−1.29	0.001	−0.62	17/20
Coma RMS	0.69 ± 0.32	0.31 ± 0.17	−0.38	<0.001	−1.16	19/20
Trefoil RMS	1.21 ± 1.36	0.48 ± 0.36	−0.73	0.014	−0.54	15/20
Spherical Aberration	0.43 ± 0.29	0.29 ± 0.20	−0.13	0.045	−0.48	14/20

## Data Availability

Due to ongoing research, the data are available upon request.

## Abbreviations

The following abbreviations are used in this manuscript:

ANOVA	Analysis of variance
CSV	Comma-separated values
D	Diopters
EBMD	Epithelial basement membrane dystrophy
FDR	False discovery rate
HOA	Higher-order aberration
IQR	Interquartile range
LMU	Ludwig Maximilian University
MAD	Median absolute deviation
OCT	Optical coherence tomography
OD	Oculus dexter (right eye)
OS	Oculus sinister (left eye)
PTK	Phototherapeutic keratectomy
RM-ANOVA	Repeated-measures analysis of variance
RMS	Root mean square
SCTK	Sequential custom therapeutic keratectomy
SD	Standard deviation
